# MicroRNA Contents in Matrix Vesicles Produced by Growth Plate Chondrocytes are Cell Maturation Dependent

**DOI:** 10.1038/s41598-018-21517-4

**Published:** 2018-02-26

**Authors:** Zhao Lin, Michael J. McClure, Junjun Zhao, Allison N. Ramey, Niels Asmussen, Sharon L. Hyzy, Zvi Schwartz, Barbara D. Boyan

**Affiliations:** 1 Department of Periodontics, School of Dentistry, Virginia Commonwealth University, Richmond, VA USA; 2 Department of Biomedical Engineering, School of Engineering, Virginia Commonwealth University, Richmond, VA USA; 3 General Dentistry, 9th People’s Hospital, College of Stomatology, Shanghai JiaoTong University School of Medicine, Shanghai, China; 4 School of Integrated Life Science, Virginia Commonwealth University, Richmond, VA USA; 5 Department of Periodontics, The University of Texas Health Science Center at San Antonio, San Antonio, TX USA; 6 Wallace H. Coulter Department of Biomedical Engineering, Georgia Institute of Technology, Atlanta, GA USA

## Abstract

Chondrocytes at different maturation states in the growth plate produce matrix vesicles (MVs), membrane organelles found in the extracellular matrix, with a wide range of contents, such as matrix processing enzymes and receptors for hormones. We have shown that MVs harvested from growth zone (GC) chondrocyte cultures contain abundant small RNAs, including miRNAs. Here, we determined whether RNA also exists in MVs produced by less mature resting zone (RC) chondrocytes and, if so, whether it differs from the RNA in MVs produced by GC cells. Our results showed that RNA, small RNA specifically, was present in RC-MVs, and it was well-protected from RNase by the phospholipid membrane. A group of miRNAs was enriched in RC-MVs compared RC-cells, suggesting that miRNAs are selectively packaged into MVs. High throughput array and RNA sequencing showed that ~39% miRNAs were differentially expressed between RC-MVs and GC-MVs. Individual RT-qPCR also confirmed that miR-122-5p and miR-150-5p were expressed at significantly higher levels in RC-MVs compared to GC-MVs. This study showed that growth plate chondrocytes at different differentiation stages produce different MVs with different miRNA contents, further supporting extracellular vesicle miRNAs play a role as “matrisomes” that mediate the cell–cell communication in cartilage and bone development.

## Introduction

Endochondral bone formation consists of a developmental cascade of chondrocyte maturation that is well-regulated in temporal and spatial dimensions^1^. This is reflected in a zonal change in cell morphology and function from resting to proliferative, prehypertrophic and hypertrophic chondrocytes followed by calcification of the extracellular matrix, vasculogenesis, and bone formation on the calcified cartilage scaffold. Central to the process of growth plate development is the role of small membrane-bound extracellular microvesicles, called matrix vesicles (MV)^2^. MVs are enriched in alkaline phosphatase specific activity compared to the plasma membrane and the first calcium phosphate crystals are observed on the inner leaflet of the MV membrane. Depending on the state of chondrocyte maturation within the growth plate, MVs have a different phospholipid composition^3^ and are selectively packed with different matrix metalloproteinases^4–6^, alkaline phosphatase activity^3,7–9^, growth factors^10^, and receptors for hormones^11–13^, suggesting they function as regulators of the endochondral bone formation environment^14–16^.

We have established a cell culture model to study the phenotypic transition of chondrocytes from the resting zone (RC) to the prehypertrophic/upper hypertrophic zone (GC), in which these two cell types are cultured separately after discarding the intervening proliferative cell zone^3^. Characterization of these two distinct cell populations has shown that they are different in morphology^3^, proliferation rate^3^, extracellular matrix components^3^, basal cation flux and membrane fluidity^17,18^, membrane phospholipid composition^3,19^, basal production of prostaglandin E_2_^20^ and production of vitamin D metabolites 1,25-dihydroxyvitamin D3 [1,25(OH)_2_D_3_] and 24,25-dihydroxyvitamin D3 [24,25(OH)_2_D_3_]^21^.

In addition, RC and GC chondrocytes exhibit marked differences in their response to vitamin D metabolites and growth factors^5,11,22^. Whereas RC cells respond primarily to 24 R,25(OH)_2_D_3_, GC chondrocytes respond primarily to 1α,25(OH)_2_D_3_. Testosterone stimulates alkaline phosphatase specific activity in GC cells, with no effects on RC cells^23^; TGF-β1 induces a dose-dependent increase in proliferation of RC cells, but this effect was not seen in GC cells^24^. Interestingly, even though RC chondrocytes will form nodules in long-term culture, these cells do not mineralize their matrix. However, bone morphogenetic protein 2 (BMP2) or 24 R,25(OH)_2_D_3_ treatment of RC cells induces a phenotypic shift into GC chondrocytes^25^, which are able to calcify their extracellular matrix.

Both types of chondrocytes produce MVs, but with distinctly different properties. MVs produced by GC cells possess greater specific activities of alkaline phosphatase (the MV marker enzyme)^3^ and phospholipase A_2_ than do MVs produced by RC cells^19^. RC MVs contain neutral metalloproteinases, whereas MVs produced by GC chondrocytes contain acidic matrix metalloproteinases^4,5,20,26^. RC MVs also have different phospholipid profiles, membrane fluidities and membrane receptors compared to MVs produced by GC cells^3,11,12,19^. Protein kinase C (PKC) activity in RC-MVs is regulated by 24 R,25(OH)_2_D_3_; however, it is stimulated by 1α,25(OH)_2_D_3_ in GC-MVs^27,28^. RC-MVs are usually not able to calcify, however, mineralization occurs in GC-MVs^29,30^. Taken together, these findings suggest that chondrocytes at different maturation status in the growth plate produce different MVs with different contents, which modulate their different local environment and satisfy different metabolic needs.

Recent studies show that microRNAs (miRNA), which are short (20–22-nucleotide), endogenous, single-stranded, non-coding RNA molecules that regulate gene expression at the post-transcriptional level^31^, are involved in endochondral bone formation^32^. miR-145 was downregulated during TGFβ3-induced chondrogenic differentiation of mesenchymal stem cells (MSCs), and targeted Sox9 mRNA transcripts^33^. miR-140 was expressed in cartilage during embryonic development, contributes to craniofacial development, and regulated histone deacetylase 4 (HDAC4), but there was no evidence to indicate its involvement in postnatal tissue^34^. miR-1 was shown to be specifically expressed in the hypertrophic zone in postnatal growth plate, and repressed HDAC4^35^. These studies substantiate the hypothesis that specific miRNA regulate growth plate cartilage.

miRNA is packaged in membrane bound vesicles termed endosomes, which are found in biofluids such as blood, plasma, urine and saliva, as well as culture medium^36^. Emerging as novel mediators in cell-cell communications, extracellular miRNA is involved in various physiological and pathological processes^37^. Recently, our lab showed that small RNA, and miRNA in particular, is highly enriched in matrix vesicles produced by GC chondrocytes^38^. Moreover, our results indicate that a group of miRNAs is selectively packed into these MVs, suggesting that MVs function as “matrisomes,” providing a mechanism for information transfer in the non-vascularized growth plate. However, it is not known whether small RNA also exists in MVs produced by RC chondrocytes and, if so, whether there is difference in their miRNA composition. The purpose of this study was to determine the presence of RNA in RC-MVs, and then use PCR array and next generation sequencing to identify their miRNA profile. We also compared the RC-MV miRNAs to those present in GC-MVs.

## Results

### Characterization of RC-MVs

**Figure 1:**
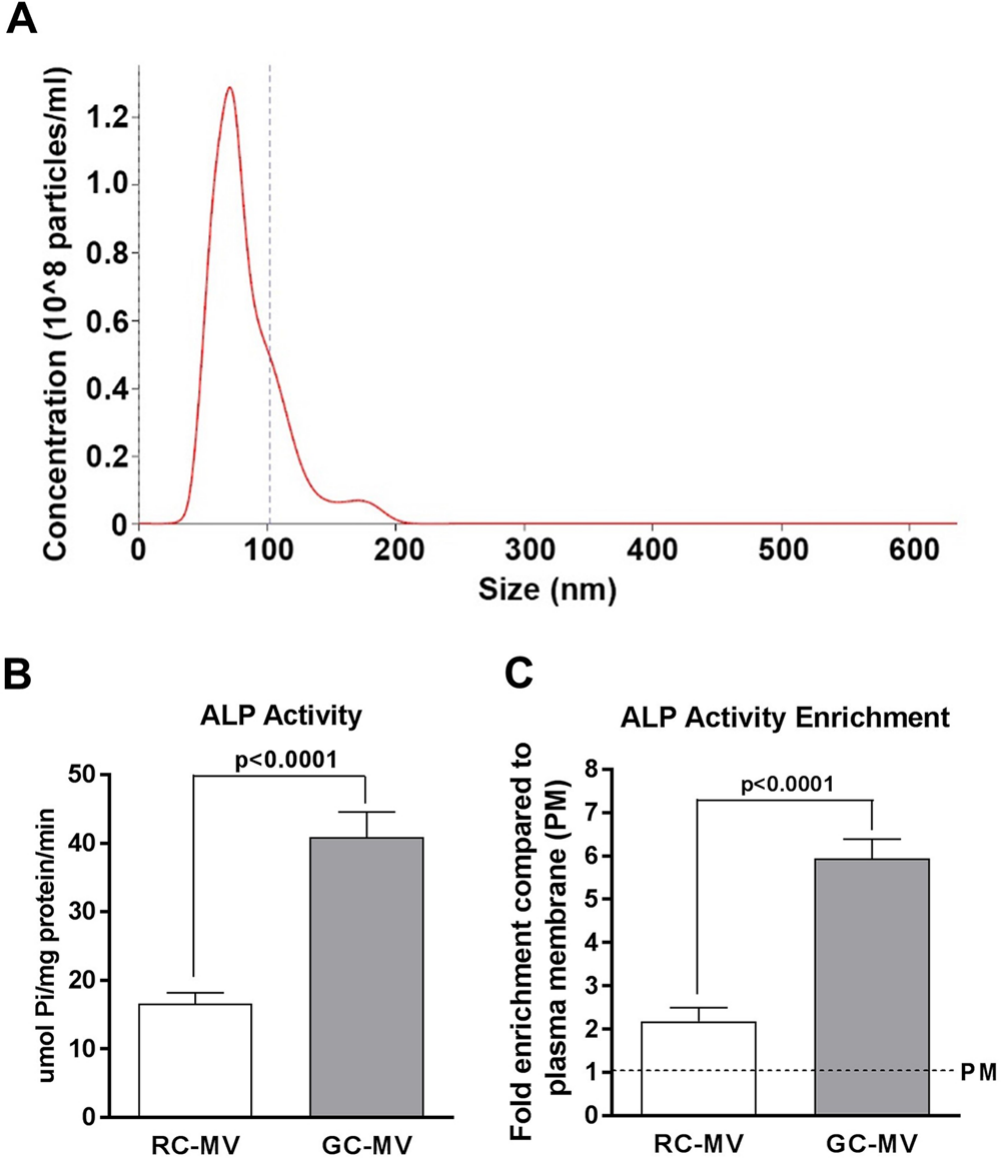
Characterization of RC-MVs. (**A**) Nanosight was used to measure the size of RC-MVs, which showed an average diameter around 80 nm. (**B**) Alkaline phosphatase (ALP) specific activity was compared between RC-MVs and GC-MVs. GC cells produced MVs with higher ALP specific activity than RC cells (n = 5 or 6). (**C**) In RC cell cultures, less ALP activity enrichment was seen in MVs compared to that in GC cell cultures (n = 5 or 6). PM=plasma membrane.

Nanosight analysis showed that MVs isolated from RC chondrocyte cultures were approximately 100 nm in diameter (Fig. 1A). These vesicles demonstrated a more than 2-fold increase in alkaline phosphatase specific activity compared to the RC plasma membrane, which is a marker for MVs (Table 1). In addition, activity in RC-MVs was lower than in MVs from GC chondrocyte cultures (Fig. 1B), which exhibited a 6-fold enrichment over plasma membrane activity (Fig. 1C), being consistent with our previous observations^3^.

**Table 1:** Alkaline phosphatase (ALP) specific activity of MVs from resting zone chondrocytes.

	ALP activity (µmol Pi/mg protein/min) (mean ± s.e.m.)	Fold enrichment
Total cell lysate	4.04 ± 0.45^c,d^	1
Mitochondria	4.67 ± 2.58^d^	1.16
Plasma membrane	8.00 ± 1.00^d,e^	1.98
MVs	16.65 ± 3.79^a,b,c,e^	4.12
Pellet from culture medium	2.33 ± 0.43^c,d^	0.57

Note: ^a^p < 0.05 compared with total cell lysate; ^b^p < 0.05 compared with mitochondria; ^c^p < 0.05 compared with plasma membrane; ^d^p < 0.05 compared with MVs; ^e^p < 0.05 compared with pellet from culture medium; n = 5 or 6. All the comparisons were done within the same cell type. (^a–e^Based on one way ANOVA). The enrichment folds compared with total cell lysate are presented on the right.

### Small RNA is selectively packaged in matrix vesicles produced by resting zone chondrocytes

**Figure 2:**
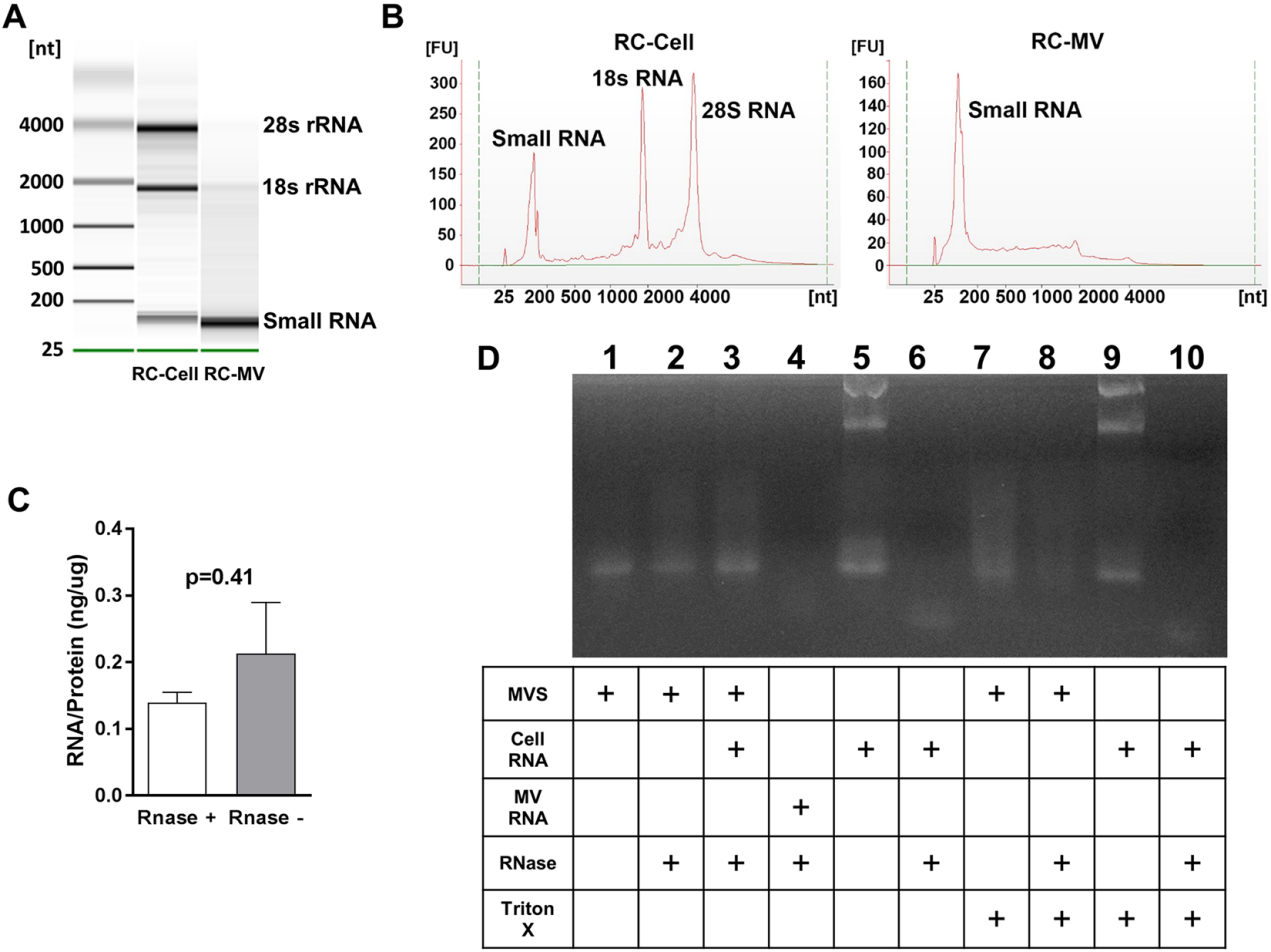
RNA exists in RC-MVs. (**A**) MV RNA was compared to cell RNA. In a 2% agarose gel, RC-MV RNA was heterogeneous in size but contained little or no large ribosomal RNA species (18S- and 28S-rRNA) compared to parent cells. Enriched small RNAs were observed in MVs. (**B**) A similar pattern was observed in the Bioanalyzer. (**C**) RC-MVs were treated by RNase A before RNA extraction by TRIzol. No significant differences in the RNA:protein ratios of MVs with or without RNase pretreatment (n = 6) were observed. (**D**) RNase was not able to digest the RNA component in MVs. However, it was able to digest the total cell RNA. When the MVs were pre-treated with the membrane detergent Triton X-100, RNA degradation was observed, suggesting that RNA in RC-MVs is protected by the intact lipid membrane.

RC cells had both large ribosomal RNA (28 s and 18 s) and small RNA, while MVs produced by these cells only exhibited small RNA that was less than 200 nt (Fig. 2A). This was confirmed by Bioanalyzer (Fig. 2B). These RNAs were packed inside MVs and were protected from enzymatic digestion by RNase. RNase treatment did not decrease the yield of RNA extracted from MVs (Fig. 2C). If the membrane was intact, RNase did not digest the MV RNA (Fig. 2D). However, when Triton X-100 was added to disrupt the membrane, RNase completely digested the RNA in the MV suspension. These results suggested that the RNA in the MVs was protected by the phospholipid bilayer membrane.

### Selective miRNAs are enriched in RC-MVs

**Figure 3:**
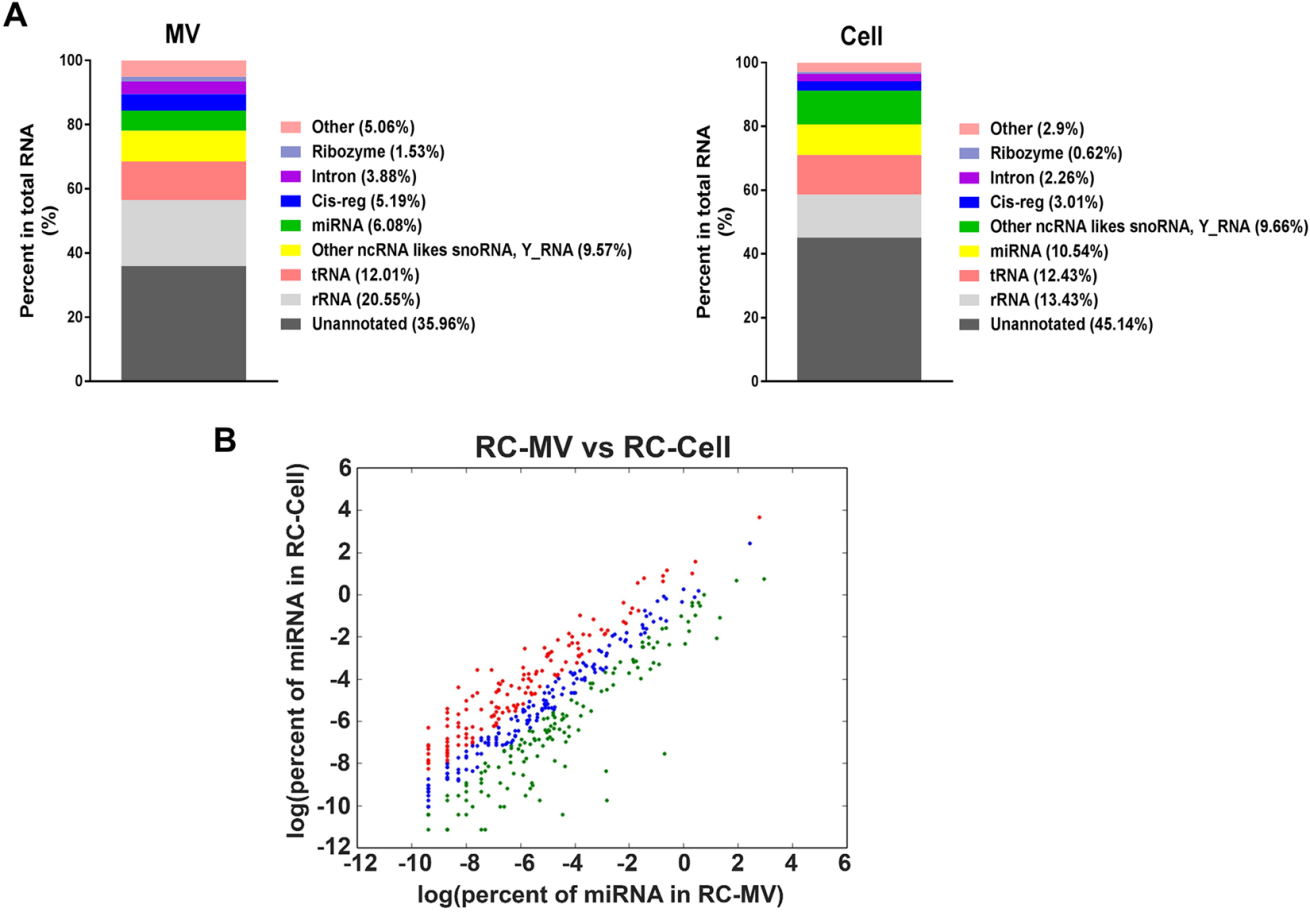
The profiles of small RNAs in RC-MVs and parent cells. (**A**) Complex populations of coding and non-coding small RNAs were found in RC-MVs in proportions that were distinct from those in the RC cells. (**B**) miRNAs in RC-MVs and RC-cells identified by RNA-seq were plotted. Red dots represent miRNAs with at least a 2-fold expression level increase in MVs than those in cells. Green dots represent miRNAs with more than a 2-fold expression level increase in cells than that in MVs. Blue indicates the overlap between these two groups. A large number of miRNAs were differentially expressed between RC-MVs and RC-cells.

We used a PCR array to determine the miRNA profile of RC-MVs, and compared that to the total cell miRNA profile. A distinct pattern was seen in RC-MVs with a selective group of miRNAs that were highly enriched (Table 2). In this data set, 13 miRNAs were only detected in RC-MVs but not RC-cells; 10 other miRNAs were found to have at least more than 2-fold enrichment in RC-MVs, which included miR-150-5p, miR-126a-3p, and miR-129-2-3p. Conversely, miRNA expression levels within the cells that were elevated compared to MVs included miR-29b-3p, miR-503-5p, and miR-27a-3p. We also obtained a global view of RC-MV small RNA using high throughput, unbiased RNA-Seq and compared the results to parent chondrocytes (Fig. 3A). The annotated small RNAs in RC-MVs included rRNA (20.55%), transfer RNA (12.01%), miRNAs (6.08%), and non-coding RNA like snoRNA, Y_RNA, etc (9.57%). In contrast, chondrocytes had significantly more miRNAs (10.54%).

**Table 2:** miRNAs with different expression levels between RC-MVs and RC-cells in miRNA PCR array.

miRNA unique in RC-MVs	miRNAs shared in RC-MVs and RC-cells		miRNA unique in RC-MVs
miRNA	miRNAs	Fold_change	miRNA
***rno-miR-451-5p***	***rno-miR-150-5p***	**10**.**23**	rno-miR-27a-3p
***rno-miR-223-3p***	***rno-miR-126a-3p***	**6**.**73**	rno-miR-219-5p
***rno-miR-142-3p***	*rno-miR-129-2-3p*	4.61	rno-miR-190a-5p
***rno-miR-122-5p***	*rno-miR-10a-5p*	3.45	rno-miR-147
*rno-miR-139-5p*	*rno-miR-125a-3p*	2.75	rno-miR-325-3p
*rno-miR-339-5p*	*rno-miR-490-3p*	2.16	rno-miR-499-5p
*rno-miR-142-5p*	*rno-miR-186-5p*	2.14	rno-miR-598-3p
*rno-miR-144-3p*	*rno-miR-196b-5p*	2.12	rno-miR-196a-5p
*rno-miR-211-5p*	*rno-miR-34b-3p*	2.11	rno-miR-298-5p
*rno-miR-133a-3p*	*rno-miR-132-3p*	2.04	rno-miR-181d-5p
*rno-miR-133b-3p*	rno-miR-136-5p	−2.07	rno-miR-540-5p
*rno-miR-200c-3p*	rno-miR-218a-5p	−2.15	rno-miR-203a-3p
*rno-miR-330-5p*	rno-miR-324-5p	−2.15	rno-miR-130b-3p
	rno-miR-193-3p	−2.18	rno-miR-384-5p
	rno-let-7i-5p	−2.38	rno-miR-384-3p
	rno-miR-29c-3p	−2.40	rno-miR-296-5p
	rno-miR-301a-3p	−2.51	rno-miR-135a-5p
	rno-miR-210-3p	−2.52	rno-miR-187-3p
	rno-miR-376a-3p	−2.54	rno-miR-291a-5p
	rno-miR-195-5p	−2.58	rno-miR-181c-5p
	rno-miR-34c-5p	−2.71	rno-miR-342-5p
	rno-miR-32-5p	−4.21	
	rno-miR-503-5p	−4.59	
	rno-miR-29b-3p	−5.65	
	rno-miR-33-5p	−5.80	
	rno-miR-34b-5p	−6.35	

Note: miRNAs that were only detected in MVs from resting zone chondrocytes were shown in the left column. miRNAs that were only detected in the resting zone chondrocytes were shown in the right column. miRNAs that were detected both in resting zone MVs and cells, but with at least a 2-fold change and p < 0.05 were shown in the middle column. miRNAs that are in Italic area: highly enriched in RC MVs. miRNAs that in underline area: mostly remained in RC cells. miRNAs highlighted in bold italic were selected for further validation by RT-PCR.

**Figure 4:**
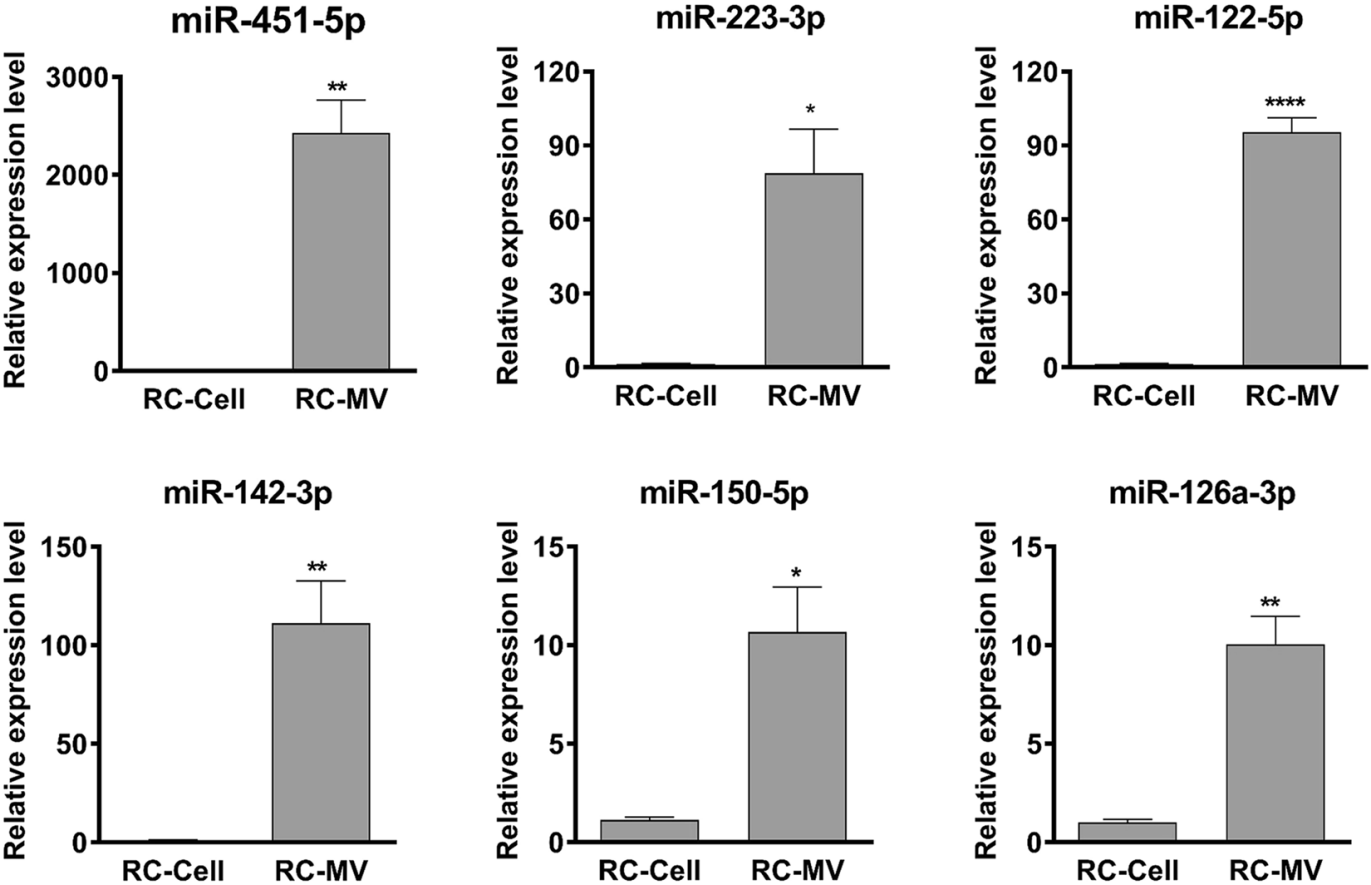
Validation of the miRNA enriched in RC-MVs compared with parent cells. Six miRNAs (miR-451-5p, miR-223-3p, miR-122-5p, miR-142-3p, miR-150-5p, miR-126a-3p) were selected for RT-qPCR assays. Consistent with previous array results, the expression of all these miRNAs were significantly higher in MVs compared to cells. These results suggest that certain miRNAs are selectively exported into MVs. *p < 0.05 in t-test. **p < 0.01 in t-test. ****p < 0.0001 in t-test (n = 3).

When the RC-MV miRNA pool was examined more closely, we determined that it was dominated by a small number of miRNAs. The most abundant miRNA in RC-MVs was miR-22-3p, which accounted for 20.27% of the total miRNA reads, followed by miR-21-5p (17.21%) and miR-143-3p (11.77%). The top 3 most abundant miRNAs occupied over 50% of the miRNA pool (Table 3), which was similar to that of the parent cell miRNA pool (Table 4). However, we also found distinct differences in the miRNA profile between RC-MVs and RC-cells (Fig. 3B). 139 (32.1%) miRNAs were found to be enriched in MVs by at least two-fold. The top 20 most RC-MV enriched miRNAs (compared to RC-cells) identified from RNA sequencing are listed in Table 5. We validated these findings using real time PCR. 6 RC-MV miRNAs (miR-451-5p, miR-223-3p, miR-122-5p, miR-142-3p, miR-150-5p, miR-126a-3p) were selected because they were only detected or were highly enriched in RC-MV based on the PCR-array (Table 2, highlighted in red). We confirmed that these miRNAs were expressed at significantly higher levels in RC-MV compared to RC-Cell (Fig. 4), suggesting that the packaging of miRNAs into resting zone chondrocyte matrix vesicles is a selective process.

**Table 3:** Top 20 most abundant miRNAs in RC-MV.

miRNA	% in RC-MVs
rno-miR-22-3p	20.27
rno-miR-21-5p	17.21
rno-miR-143-3p	11.77
rno-miR-127-3p	6.84
rno-miR-320-3p	3.50
rno-miR-134-5p	3.43
rno-miR-541-5p	1.93
rno-let-7c-5p	1.88
rno-miR-152-3p	1.77
rno-let-7b-5p	1.62
rno-let-7i-5p	1.54
rno-miR-381-3p	1.50
rno-miR-100-5p	1.49
rno-miR-99b-5p	1.36
rno-miR-370-3p	1.27
rno-miR-181a-5p	1.26
rno-miR-341	1.22
rno-miR-148a-5p	1.12
rno-miR-125b-5p	1.03
rno-miR-140-3p	0.95

**Table 4:** Top 20 most abundant miRNAs in RC-cells.

miRNA	% in RC-Cells
rno-miR-21-5p	42.70
rno-miR-143-3p	11.21
rno-let-7i-5p	5.00
rno-miR-27b-3p	3.11
rno-miR-148a-3p	2.67
rno-let-7f-5p	2.35
rno-miR-22-3p	2.23
rno-miR-199a-5p	2.07
rno-miR-127-3p	1.73
rno-miR-199a-3p	1.46
rno-miR-125b-5p	1.34
rno-let-7c-5p	1.34
rno-miR-24-3p	0.96
rno-miR-100-5p	0.91
rno-miR-26a-5p	0.91
rno-miR-30a-5p	0.79
rno-miR-379-5p	0.77
rno-miR-152-3p	0.75
rno-let-7g-5p	0.73
rno-miR-99b-5p	0.67

**Table 5:** Top 20 selectively packaged miRNAs in MVs in RNA sequencing.

miRNA	% in RC-MVs	% in RC-Cells	Fold enrichment in MVs
rno-miR-122-5p	4.01E − 01	4.91E − 04	816.82
rno-miR-451-5p	6.14E − 02	7.67E − 05	800.54
rno-miR-144-3p	1.30E − 02	3.07E − 05	423.20
rno-miR-23a-5p	6.40E − 03	1.23E − 04	52.16
rno-miR-320-3p	3.50E + 00	1.29E − 01	27.10
rno-miR-490-3p	9.02E − 03	3.84E − 04	23.51
rno-miR-615	1.57E − 02	1.23E − 03	12.78
rno-miR-296-3p	1.75E − 02	1.41E − 03	12.39
rno-miR-365-5p	1.33E − 02	1.12E − 03	11.92
rno-miR-134-5p	3.43E + 00	2.88E − 01	11.90
rno-miR-664-2-5p	1.37E − 02	1.18E − 03	11.60
rno-miR-770-3p	8.78E − 02	8.15E − 03	10.78
rno-miR-148a-5p	1.12E + 00	1.04E − 01	10.76
rno-miR-129-5p	1.91E − 01	1.97E − 02	9.72
rno-miR-22-3p	2.03E + 01	2.23E + 00	9.10
rno-miR-666-5p	1.14E − 02	1.30E − 03	8.71
rno-miR-219a-1-3p	7.85E − 03	9.82E − 04	7.99
rno-miR-423-5p	3.24E − 01	4.09E − 02	7.94
rno-miR-760-3p	2.24E − 02	2.98E − 03	7.51
rno-miR-127-5p	5.82E − 01	8.26E − 02	7.05

### Functional annotation of RC-MV enriched miRNAs

miRNAs function by binding to target mRNAs and inhibiting their translation or promoting degradation. We identified the target genes of the top most enriched miRNA in RC-MVs and analyze the signaling pathways that are potentially involved. Based on results from KEGG signaling pathway analysis, key functions of target genes included roles in stem cell regulation, Hippo signaling, Wnt signaling, Rap1 signaling, ErbB signaling, focal adhesion, and cell cycle, many of which have been shown to be involved in chondrocyte proliferation and differentiation (Table 6).

**Table 6:** KEGG pathways targeted by the top 10 RC-MV enriched miRNAs.

KEGG pathway	*P* value
Axon guidance	2.30E − 13
Signaling pathways regulating pluripotency of stem cells	6.70E − 13
Hippo signaling pathway	2.60E − 12
Wnt signaling pathway	3.20E − 09
Focal adhesion	6.80E − 09
MAPK signaling pathway	1.30E − 08
Thyroid hormone signaling pathway	3.50E − 08
PI3K-Akt signaling pathway	1.40E − 07
Rap1 signaling pathway	1.60E − 07
TGF-beta signaling pathway	3.00E − 07
cAMP signaling pathway	7.40E − 07
ErbB signaling pathway	8.20E − 07
cGMP-PKG signaling pathway	2.30E − 06
Calcium signaling pathway	2.50E − 06
Endocytosis	3.50E − 06
Oxytocin signaling pathway	3.60E − 06
Neurotrophin signaling pathway	4.30E − 06
Ras signaling pathway	5.90E − 06
Circadian entrainment	7.80E − 06
Insulin resistance	1.40E − 05
Dorso-ventral axis formation	1.70E − 05

### MV miRNA component is dependent on the cell maturation status

**Figure 5:**
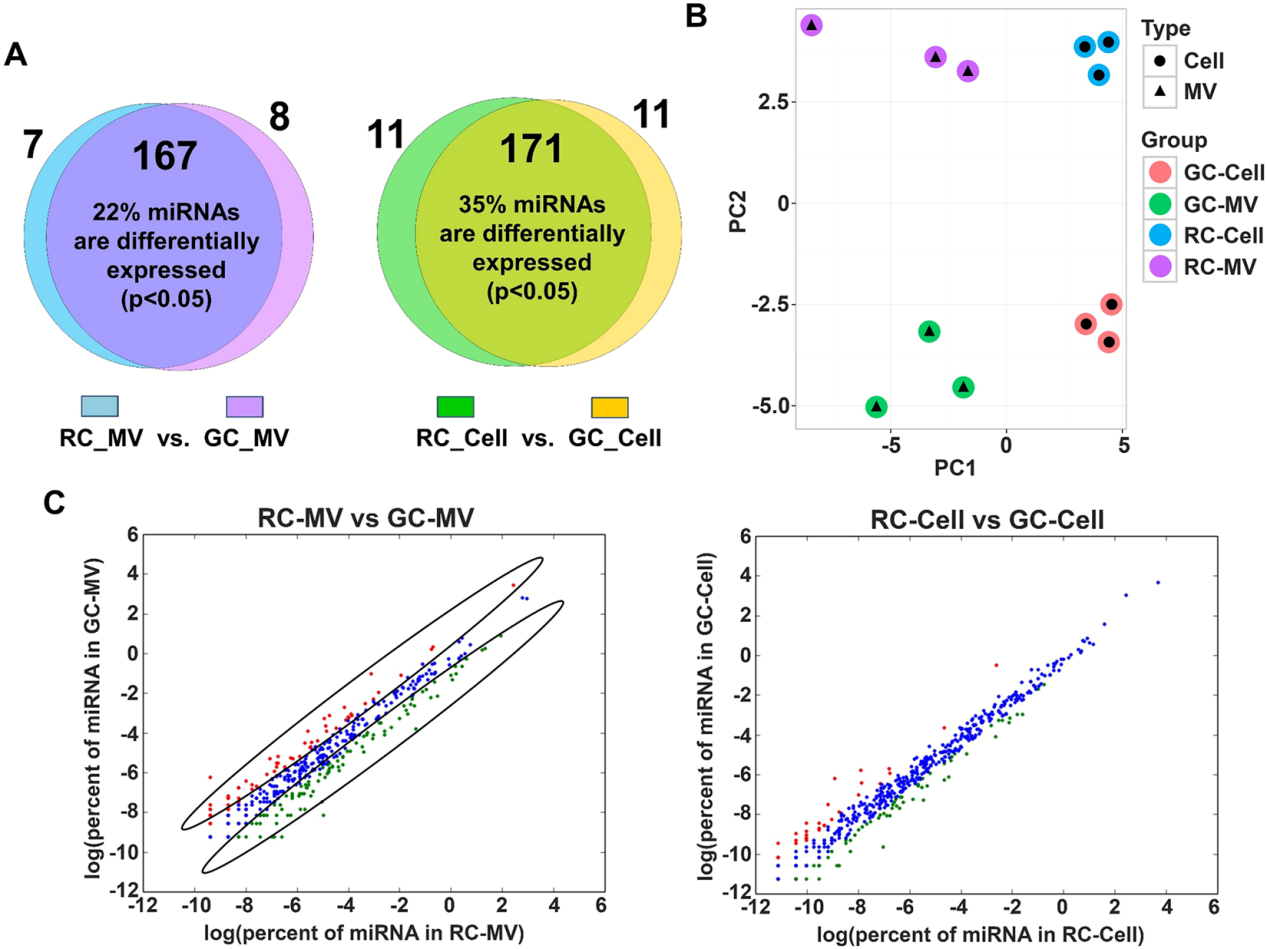
Distinct differences in the miRNAs between RC-MVs and GC-MVs. (**A**) Different miRNAs patterns were identified from PCR-array between two maturation status of chondrocytes, both in MVs and the parent cells. (**B**) Principal component analysis (PCA) of the miRNA PCR-array data for all the four different types of samples: RC-MVs, RC-cells, GC-MVs and GC-cells. The differences among these samples were clear, as larger difference showed on the x-axis direction compared to that on the y-axis. This suggests that the differences between MVs and cells were larger than between two different cell maturation stages. (**C**) Left panel: miRNAs in RC-MVs and GC-MVs identified by RNA-seq were plotted. Red dots represent miRNAs with at least a 2-fold expression level increase in GC-MVs than in RC-MVs. Green dots represent miRNAs with a more than 2-fold expression level increase in RC-MVs than in GC-MVs. Blue indicates the overlap between these two groups. A large number of miRNAs were differentially expressed between RC-MVs and GC-MVs. Right panel: similar comparison was done between RC-cells and GC-cells. Interestingly, the difference between RC-cells and GC-cells was not as large as that was seen between RC-MVs and GC-MVs.

We have previously shown distinct differences in MVs that can be ascribed to the level of chondrocyte maturation. We recently reported that GC-MVs were packed with selective miRNAs. Based on these studies, we hypothesized that miRNAs were selectively packed into RC and GC MVs. To investigate unique maturation-dependent differences, we compared the PCR array results of RC-MVs to those of GC-MVs. In the 182 miRNAs detected, 10 miRNAs were identified in GC-MVs and 7 were found in RC-MVs (Fig. 5A). 35 miRNAs were differently expressed between RC-MVs and GC-MVs with at least a more than 2-fold difference (increase or decrease) (Table 7). We also identified a group of miRNAs that showed different expression levels between RC cells and GC cells (Table 8). Using principle component analysis, we observed differences between RC-MVs (purple dots, top left) and GC-MVs (green dots, bottom left) (Fig. 5B). While 39% of miRNAs showed differential expression level with more than 2-fold difference between RC-MVs (green dots, circled) and GC-MVs (red dots, circled), we observed a unique separation between these miRNA (Fig. 5C), further supporting our hypothesis. Therefore, the selective packaging of miRNAs into MVs exhibits spatial and maturation dependence. Interestingly, less miRNAs were found differentially expressed between RC-cells and GC-cells than between RC-MVs and GC-MVs (Fig. 5C), suggesting more heterogeneous miRNA profiles exist in the extracellular vesicles than that in the cytoplasm. It is worth mentioning that the difference between RC-MVs and GC-MVs was less dramatic than between RC-MVs and RC-cells or between GC-MVs and GC-cell, since the distance at the x-axis represents the most prominent difference followed by the distance at the y-axis. This indicates that the difference between MVs and the cells that produce them is more significant than that between two cell maturation statuses (RC vs. GC) (Fig. 5C).

**Table 7:** miRNAs with different expression levels between RC-MVs and GC-MVs in miRNA PCR array.

miRNA unique in RC-MVs	miRNAs shared in RC-MVs and GC-MVs		miRNA unique in GC-MVs
miRNA	miRNAs	Fold_change	miRNA
**rno-miR-380-5p**	**rno-miR-503-5p**	**8.73**	rno-miR-325-3p
**rno-miR-200c-3p**	**rno-miR-672-5p**	**4.31**	rno-miR-326-3p
**rno-miR-874-3p**	**rno-miR-450a-5p**	**3.85**	rno-miR-219-5p
**rno-miR-339-3p**	**rno-miR-204-5p**	**3.10**	rno-miR-298-5p
**rno-miR-340-3p**	**rno-miR-322-5p**	**2.78**	rno-miR-130b-3p
**rno-miR-186-5p**	**rno-miR-668**	**2.78**	rno-miR-760-3p
**rno-miR-212-3p**	**rno-miR-362-5p**	**2.37**	rno-miR-598-3p
	**rno-miR-125a-3p**	**2.37**	rno-miR-181c-5p
	**rno-miR-329-3p**	**2.29**	rno-miR-592
	**rno-miR-181b-5p**	**2.14**	rno-miR-499-5p
	rno-miR-130a-3p	−2.01	
	rno-miR-129-5p	−2.05	
	rno-miR-20a-5p	−2.11	
	rno-miR-133a-3p	−2.17	
	rno-miR-365-3p	−2.29	
	rno-miR-18a-5p	−2.30	
	rno-miR-143-3p	−2.39	
	rno-miR-150-5p	−2.40	
	rno-miR-195-5p	−2.48	
	rno-miR-451-5p	−2.55	
	rno-miR-497-5p	−2.57	
	rno-miR-223-3p	−2.57	
	rno-miR-582-5p	−2.60	
	rno-miR-132-3p	−2.63	
	rno-miR-133b-3p	−2.65	
	rno-miR-29b-3p	−2.74	
	rno-miR-192-5p	−2.76	
	rno-miR-126a-3p	−2.81	
	rno-miR-210-3p	−2.83	
	rno-miR-122-5p	−2.88	
	rno-miR-142-5p	−3.07	
	rno-miR-142-3p	−3.57	
	rno-miR-139-5p	−3.69	
	rno-miR-144-3p	−4.78	
	rno-miR-10a-5p	−5.93	

Note: miRNAs that were only detected in MVs from RC were shown in the left column. miRNAs that were only detected in MVs from GC were shown in the right column. miRNAs that were detected in MVs from both RC and GC cells, but with at least a 2-fold change and p < 0.05 were shown in the middle column. miRNAs that are bold area: highly enriched in RC-MVs. miRNAs that in underline area: mostly enriched in GC-MVs.

**Table 8:** miRNAs with different expression levels between RC-cells and GC-cells in miRNA PCR array.

miRNA unique in RC-cells	miRNAs shared in RC-cells and GC-cells		miRNA unique in GC-cells
miRNA	miRNAs	Fold_change	miRNA
**rno-miR-203a-3p**	**rno-miR-503-5p**	**4.62**	rno-miR-339-5p
**rno-miR-384-5p**	**rno-miR-204-5p**	**4.31**	rno-miR-139-5p
**rno-miR-135a-5p**	**rno-miR-380-5p**	**3.64**	rno-miR-211-5p
**rno-miR-335**	**rno-miR-450a-5p**	**3.60**	rno-miR-760-3p
**rno-miR-384-3p**	**rno-miR-322-5p**	**3.07**	rno-miR-592
**rno-miR-291a-5p**	**rno-miR-542-5p**	**2.69**	rno-miR-142-3p
**rno-miR-27a-3p**	**rno-miR-376c-3p**	**2.02**	rno-miR-330-5p
**rno-miR-147**	rno-miR-143-3p	−2.21	rno-miR-182
**rno-miR-339-3p**	rno-miR-449a-5p	−2.27	rno-miR-326-3p
**rno-miR-484**	rno-miR-17-5p	−2.27	rno-miR-141-3p
	rno-miR-497-5p	−2.38	rno-miR-206-3p
	rno-miR-132-3p	−2.54	
	rno-miR-296-5p	−2.69	
	rno-miR-196b-5p	−2.73	
	rno-miR-582-5p	−4.06	
	rno-miR-298-5p	−4.85	
	rno-miR-10a-5p	−10.69	

Note: miRNAs that were only detected in RC cells were shown in the left column. miRNAs that were only detected in GC cells were shown in the right column. miRNAs that were detected in cells from both RC and GC, but with at least a 2-fold change and p < 0.05 were shown in the middle column. miRNAs that are in bold area: highly enriched in RC-cells. miRNAs that in underline area: mostly enriched in GC-cells.

**Figure 6:**
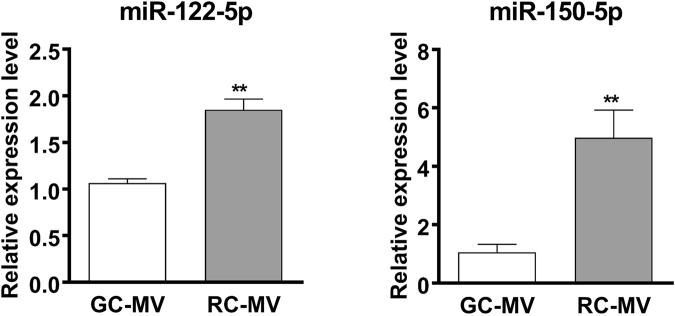
RT-qPCR was used to further validate the differences in miRNA expression between RC-MVs and GC-MVs. miR-122-5p and miR-150-5p had significantly higher expression levels in RC-MVs than GC-MVs, suggesting the miRNAs contents in MVs are associated with the cell maturation status. **p < 0.01 in t-test. (n = 6).

To further validate our observations in miRNA PCR-array and RNA-Seq, we selected a set of miRNAs that are highly expressed in RC-MVs and performed RT-qPCR to compare their expression levels between RC-MVs and GC-MVs. Although we did not observe significant differences in some of the selected miRNAs such as miR126a-3p and miR-451-5p, higher expression levels of miR-122-5p and miR-150-5p were consistently seen in RC-MVs compared with GC-MVs (Fig. 6). This further suggests that cells at different maturation status produced different matrix vesicles with different miRNA contents.

## Discussion

In this study, we demonstrate that RNA exists in MVs produced by costochondral growth plate resting zone cartilage cells. This RNA is packaged by the cells within the extracellular matrisomes and is protected by the MV membrane. RNase treatment of intact RC MVs didn’t digest their RNA component, however significant degradation occurred after the membrane was disrupted by Triton X-100. Moreover, a set of miRNAs was specifically enriched in the MVs, further supporting the hypothesis that their inclusion involves a regulated process.

A variety of RNA species were found in RC-MVs, similar to RNA species found in MVs produced by GC chondrocytes^38^. The majority of the RNA pool in RC-MVs was rRNA and tRNA. It is still not clear the biological meaning of these RNAs in the regulation of extracellular matrix and cell behavior. Recently, it was reported that tRNAs can be cleaved into small fragments that can also function as miRNAs^39^. Despite these similarities, however, PCR array and RNA-Seq showed that a large group of miRNAs was differentially expressed in MVs from RC and GC cells. Individual RT-PCR confirmed that there were significant differences in some miRNAs, such as miR-122-5p and miR-150-5p.

Chondrocytes at different maturation states in the growth plate not only produce zone-specific extracellular matrix^3,40–42^, but as noted above, their matrix vesicles differ in phospholipid profile^3^, enzyme content^3–5^, and response to hormones^11,20,28^. Our results add to this by demonstrating maturation-dependent differences in miRNA composition. This suggests that MV miRNAs may be involved in the regulation of zone-specific phenotypic behavior of the cells. Our previous observation that release of matrix vesicle contents is mediated by regulated secretion of 1α,25(OH)_2_D_3_ and 24R,25(OH)_2_D_3_ and their interaction with the MV membrane^5,43–45^, provides a mechanism for modulating the bioavailability of the miRNAs during endochondral development.

The relative differences between miRNA in RC and GC cells and the differences between RC-cells and RC-MVs or GC-cells and GC-MVs were greater than the differences between RC-MVs and GC-MVs. Chondrocytes in the resting zone of the growth plate will eventually express a growth zone phenotype, so it is likely that the phenotypic shift involves only a subset of components. MV species are heterogeneous^46^, so it is also possible that not all MVs are uniformly modulated during growth plate development.

Further investigation is needed to determine whether there are general properties that define matrisomes or if they are tissue or cell specific. Others have reported the presence of RNA in the extracellular matrix of mineralizing tissues^47^, and miRNAs have been found in dermis, bladder and small intestinal submucosa, which are associated with extracellular matrix-bound nanovesicles^48^. Compared to other extracellular vesicles such as exosomes, MVs are unique in many ways. MVs have a strong affinity to collagen and stay in the extracellular matrix, while exosomes are released into the culture media^3,38^. The biogenesis mechanisms of MVs and exosomes are different as well. MVs are formed by a cell “budding” mechanism, but exosomes are formed through membrane invagination^49^. MVs released by resting zone and growth zone chondrocytes also express higher alkaline phosphatase activity than the plasma membrane and compared to membrane vesicles they secrete into the medium^3^. Furthermore, the heterogeneity of matrix vesicles produced by chondrocytes^46^, indicates that they may perform multiple functions in the extracellular matrix. A recent study also found that mesenchymal stem cells secret at least three different types of microvescicles with different RNA material^50^, raising the possibility that miRNAs may be differentially packaged in subsets of matrisomes and exosomes to modulate specific cell functions.

Extracellular miRNAs, exosomal miRNAs in particular, have been shown to play an important role in cell–cell communication in physiological and pathological processes^37^. Similarly, MV miRNAs may also function as a modulator during chondrogenesis by transferring to other cells in the local environment in an autocrine or paracrine manner, and thereby, regulating their gene expression and biological behavior. Functional analysis suggests that MV miRNAs are associated with stem cell regulation. miR-122-5p, which has been related to liver function and HCV infection^51^, was more highly expressed in RC-MVs than GC-MVS. Others have shown that miR-122-5p targets the IGF-1R signaling pathway, which is a major player in the musculoskeletal system^52^. Our preliminary data showed that overexpression of miR-122-5p enhanced chondrocyte proliferation and maintained the stemness of these cells (data not shown). miR-150-5p, which is also higher in RC-MVs was reported to target matrix metalloproteinases^53,54^. Therefore, RC-MV enriched miRNAs may help maintain the local environment needed for RC cells to maintain a stronger proliferative capability than GC cells.

In conclusion, we demonstrated that RNA is present in MVs derived from resting zone chondrocytes, and selective miRNAs are enriched. We also observed that miRNAs are differentially expressed between RC-MVs and GC-MVs, although these differences are not as prominent as those between RC-MVs and RC-cells. Our results further suggest the importance of extracellular matrix RNA during chondrogenesis and endochondral bone formation.

## Methods

### Chondrocyte cultures

Chondrocytes were isolated by enzymatic digestion of the costochondral cartilage of 125 g male Sprague Dawley rats and cultured as described by Boyan *et al*. previously^3^. All procedures conducted on animals followed a protocol approved by the Institutional Animal Care and Use Committee (IACUC) at Virginia Commonwealth University, and all experiments were performed in accordance with relevant guidelines and regulations. Rib cages were removed and placed in Dulbecco’s modified Eagle’s medium (DMEM, Life Technologies). The resting zone and growth zone cartilage was carefully dissected out, sliced, and incubated in 1% trypsin (Life Technologies) for 1 hour and 0.02% collagenase for 3 hours. After filtering by 40-micro nylon mesh, cells were collected by centrifugation, resuspended in DMEM, and plated at a density of 25,000 cells/cm^2^. Fetal bovine serum (FBS, Life Technologies) was centrifuged at 184,000 × g for 4 h to remove the bovine serum exosomes. Chondrocytes were incubated in DMEM containing 10% exosome-free FBS, 1% penicillin/streptomycin and 50 mg/ml ascorbic acid (Sigma-Aldrich). Cell culture medium was changed 24 hours after plating and then at 72-hour intervals. At confluence (5–7 days), cells were subcultured using the same plating densities and allowed to return to confluence. All experiments used confluent cells in their fourth passage.

### MV isolation and characterization

MVs were isolated from chondrocyte cultures by differential centrifugation of the trypsin digested cell layer, as described in detail previously^3^. Briefly, after trypsinization (0.25% Trypsin-EDTA, Life Technologies) for 10 min, 1 × DPBS containing 10% exosome-free FBS was added to stop the reaction. The MVs were isolated by sequential centrifugation at 500 g for 5 min, 21,000 g for 20 min, and 184,000 g for 70 min. The MV pellets were washed with 0.9% NaCl and recentrifuged at 184,000 g for 70 min. The same centrifugation protocol was used to pellet the microvesicles in cell culture media. Alkaline phosphatase specific activity was measured and normalized to protein content. In addition, to validate that MVs were isolated but not plasma membrane debris, plasma membranes were isolated from the cell pellet as described previously and alkaline phosphatase activity determined. Alkaline phosphatase specific activity was enriched in MVs at least 2 folds compared to plasma membrane, which was consistent with our previous studies. The original MVs solution was diluted 200 times for nanoparticle tracking analysis (Nanosight, Malvern, UK).

### RNA extraction and detection

RNA was isolated from MVs or cells using TRizol (Life Technologies). RNA was eluted with RNase-free water and quantified by NanoDrop spectrophotometer (Thermo Scientific). For agarose gels, 100 ng RNA was run and visualized on a 2.2% agarose gel FashGel^TM^ System (Lonza). Bioanalyzer analyses were performed using 300 ng RNA with an RNA 600 Nano Kit (Agilent) and an Agilent 2100 Bioanalyzer (Agilent) according to manufacturer’s protocol. To confirm that RNA was inside and not outside the MVs, the vesicles were treated with RNase A (10 mg/ml, Qiagen) before RNA extraction as described previously^38^. MVs were also incubated with 0.05% Triton-X 100 in order to break down the membrane in some experiments.

### miRNA PCR profiling in MVs

cDNA library preparation and miRNA profiling were performed in MV and cell RNA from RC and GC chondrocytes as described previously^38^. The microRNA PCR array (miRCURY LNA Universal RT microRNA PCR array) included 223 rat miRNAs. Details in data normalization and analysis were provided in supplementary materials.

### Reverse transcription and quantitative real-time PCR (RT-qPCR)

50 ng RNA was reverse transcribed to cDNA with miScript II RT kit (Qiagen), followed by real-time qPCR using the StepOnePlus Real-time PCR System and miScript SYBR Green PCR Kit (Qiagen). Fluorescence values were quantified as starting quantities using known dilutions of standard control. The reverse primer was the universal primer in the kit. The sequences for the forward primers were synthesized by Eurofins MWG Operon: rno-miR-451-5p: AAACCGTTACCATTACTGAGTT; rno-miR-223-3p: TGTCAGTTTGTCAAATACCC; rno-miR-122-5p: TGGAGTGTGACAATGGTGTT; rno-miR-142-3p: TGTAGTGTTTCCTACTTTATGGA; rno-miR-150-5p: TCTCCCAACCCTTGTACCAGT; rno-miR-126a-3p: TCGTACCGTGAGTAATAATGCG; rno-miR-652-3p: AATGGCGCCACTAGGGTT.

### RNA sequencing (RNA-Seq)

RNA was isolated from MVs or cells using TRizol. Next generation sequencing was performed in an Illumina HiSeq. 2500 (Illumina, San Diego, CA). Data normalization and analysis were conducted with the miARma-Seq tool (see supplemental materials). Briefly, sequence quality was assessed with FASTQC; sequence reads were aligned with Bowtie2 to the NCBI *Rattus norvegicus* annotation release 105 (Rnor_6.0). The resulting bam files were used by miARma-Seq for tabulating discovered miRNA. Reads were also aligned to Rfam 12.3 with Bowtie2 for categorization of RNA reads. To quantify the differential expression of miRNA between samples, the count of each miRNA was normalized as the percent of all discovered miRNAs in that sample. The fold-change was calculated as the percent expression of MV miRNA/Cell miRNA. KEGG Pathway enrichment analyses were performed based on the target genes of selected miRNAs.

### Statistical analysis

Alkaline phosphatase specific activity is presented as mean ± standard error of the mean (SEM) for 6 independent MV samples per variable. RT-qPCR is presented as mean ± SEM for 3 independent samples per variable. Data were examined by ANOVA with post-hoc Bonferroni’s modification of Student’s t-test. A p value of less than 0.05 was considered statistically significant. Statistical analyses for miRNA array and RNA-Seq data were performed according to our previous protocol^38^.

### Data availability statement

The datasets generated during the current study have been submitted to NCBI GEO database (GSE106348).

## Electronic supplementary material

Supplementary Materials

Supplementary data_PCR-array
