# Supplementary material for: MicroRNA Contents in Matrix Vesicles Produced by Growth Plate Chondrocytes are Cell Maturation Dependent
Source: Sci Rep. 2018 Feb 26;8:3609. doi: 10.1038/s41598-018-21517-4 (PMC5826934; doi:10.1038/s41598-018-21517-4)
Supplement: Supplementary file 1 — Supplementary Materials [file 41598_2018_21517_MOESM1_ESM.doc]

**SUPPLEMENTARY MATERIALS**

**Title**: MicroRNA Contents in Matrix Vesicles Produced by Growth Plate Chondrocytes Are Cell Maturation Dependent

**Authors**: Zhao Lin^1,2^, Michael J. McClure^2^, Junjun Zhao^1,2,3^, Allison N. Ramey^2^, Niels Asmussen^4^, Sharon L. Hyzy^2^, Zvi Schwartz^2,5^, Barbara D. Boyan^2,6*^

**Affiliations**: ^1^Department of Periodontics, School of Dentistry, Virginia Commonwealth University, Richmond, VA; ^2^Department of Biomedical Engineering, School of Engineering, Virginia Commonwealth University, Richmond, VA; ^3^General Dentistry, 9th People's Hospital, College of Stomatology, Shanghai JiaoTong University School of Medicine, Shanghai, China; ^4^School of Integrated Life Science, Virginia Commonwealth University, Richmond, VA; ^5^Department of Periodontics, The University of Texas Health Science Center at San Antonio, San Antonio, TX; ^6^Wallace H. Coulter Department of Biomedical Engineering, Georgia Institute of Technology, Atlanta, GA

**Key Words**: matrix vesicles, miRNA, cell membrane, growth plate chondrocyte, exosomes, matrisomes, endochondral calcification, differentiation

***Corresponding Author**:

Barbara D. Boyan, Ph.D.

School of Engineering

Virginia Commonwealth University

601 W. Main Street

Richmond, VA 23284 USA

Tel.: +1 804 828 0190

E-mail: [bboyan@vcu.edu](mailto:bboyan@vcu.edu)

**1. miRNA PCR array**

**1.1 Summary of miRNA PCR array**

The miRNA PCR array was performed using the miRCURY LNA Universal RT microRNA PCR array platform (Exiqon). A rigorous and automated PCR data QC pipeline was used to ensure the assay quality. The panel profiling was successfully completed. The raw data all show good data quality. **Supplementary Table 1** summarizes the number of microRNAs for the project. **Supplementary Figure 1** shows the number of microRNAs detected in our samples as well as the Cq value of the global mean for each of the samples.

|  | Number of assays |
| --- | --- |
| miRCURY LNA Universal RT microRNA PCR Mouse&Rat panel I | 372 |
| Assays with signal in all samples | 146 |
| Average number of assays detected per sample | 185 |


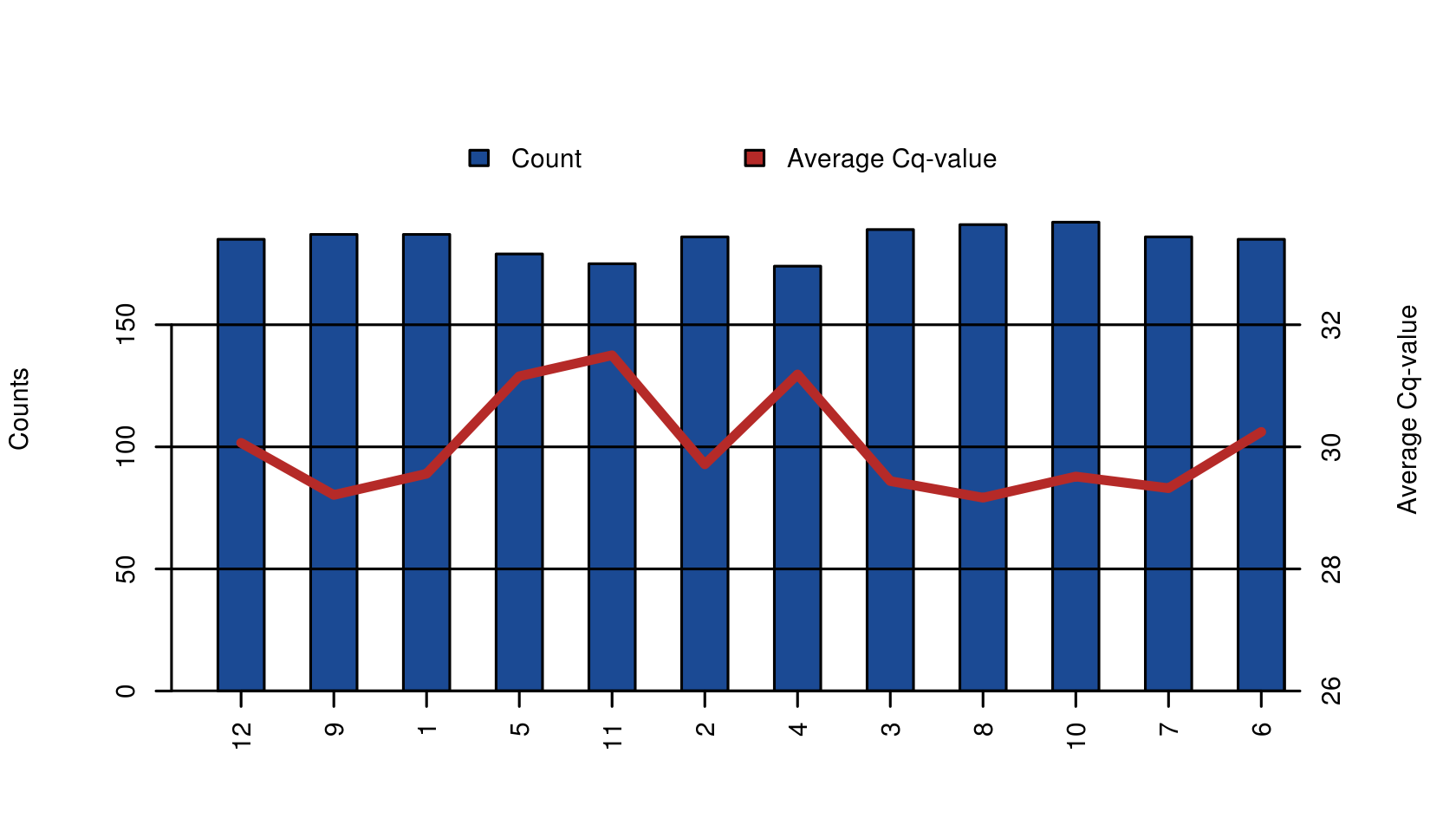
**Supplementary Table 1**: The above table lists the number of assays present of the miRCURY LNATM Universal RT microRNA PCR panels used for this study, and the number of assays that were detectable in all of your samples is listed.

**Supplementary Figure 1**: Graphical illustration of the microRNA content. The blue bars represent umber of microRNAs detected and the red line shows the average Cq value for the commonly expressed microRNAs. On average, 185 microRNA were detected per sample. X-axis shows the sample numbers.

**1.2 Data Quality Control**

Each individual amplification product on the PCR panels was scrutinized by: 1) melting curve analysis; 2) calculation of amplification efficiency; and 3) comparison of Cq value to background level in the negative control sample. Data that derive from PCR reactions that fall outside our thresholds and specifications (see details below) are flagged and removed. This ensures that the data analysis is performed with a consistent high quality data set.

**Melting curve analysis**

An additional step in the real-time PCR analysis was performed to evaluate the specificity of the amplification products by generating a melting curve for each reaction. The appearance of a single peak with the expected Tm is an indication that a single specific product was amplified during the qPCR process. PCR reactions that gave rise to multiple melting curve peaks or single peaks with melting temperature that was inconsistent with the specifications for the corresponding assay (in-house database) were flagged and removed from the dataset. **Supplementary Figure 2** is an example of SampleSubmissionPlot and the conclusion of the melting curve analysis is shown. Flagged reactions are marked with crosses.

**
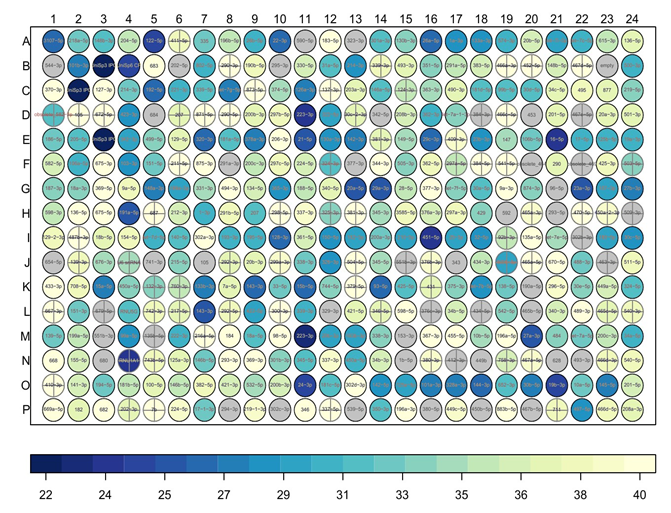
**

**Supplementary Figure 2**: Example of SampleSubmissionPlot illustrating the Cqs obtained for each well in a 384-well plate. The color scale indicates the level of expression (Cq). The circles with crosses indicate assays that have more than one peak on the melting curve, or that have a Tm that deviate from the in-house database of Tm-values.

**Signals from negative controls**

A “no template” sample in the RT step was included as a negative control. This type of negative control is used as this is the most stringent type of control applicable. It will detect RNA contamination in the RT step. An assay detected 5 Cqs lower than the negative control was included in the data analysis. For assays that do not yield any signal on the negative control, the upper limit of detection is set to Cq=37.


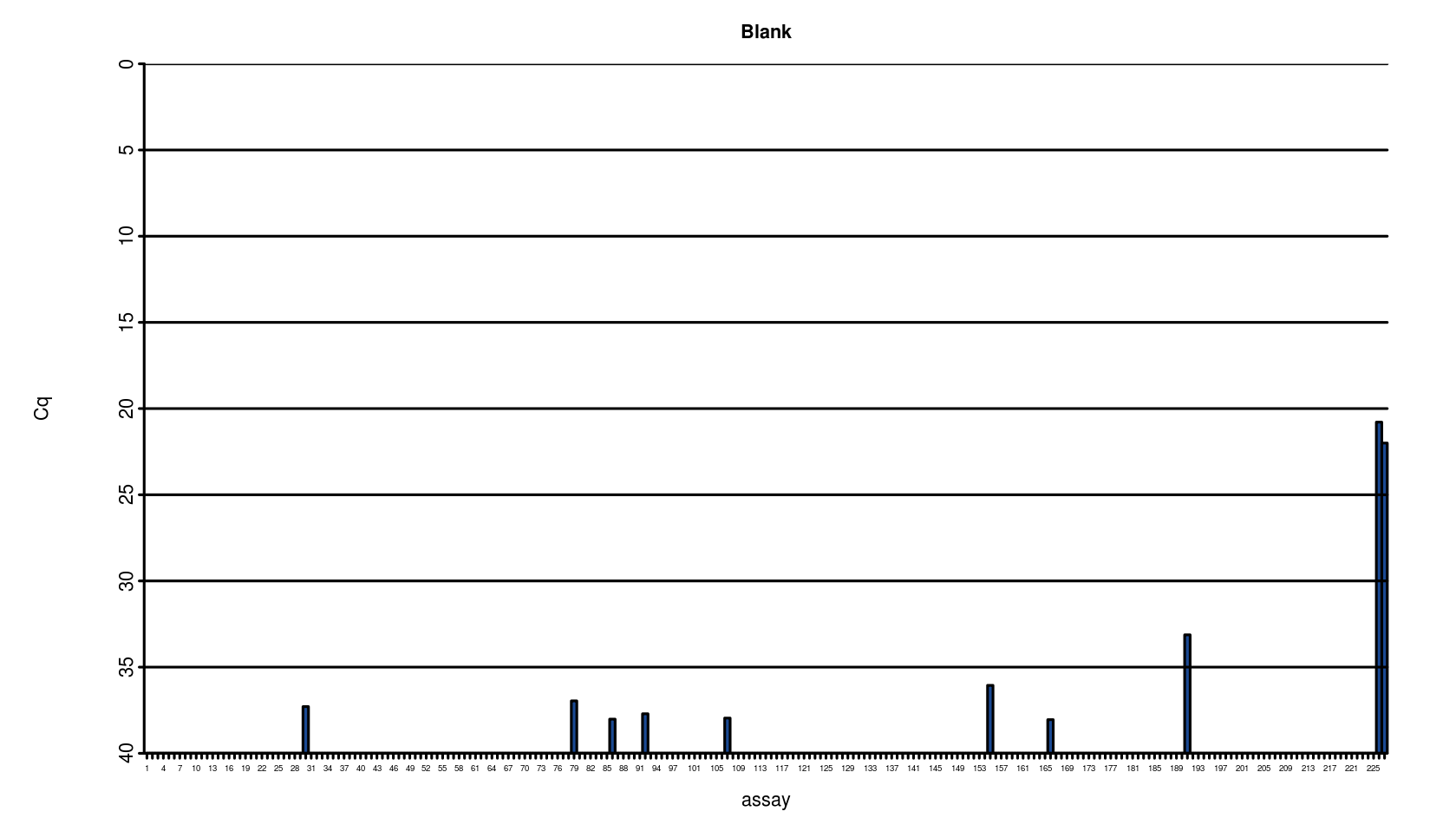


**Supplementary Figure 3**: Bar diagram showing raw Cq values for the negative control sample. Please notice that the positive controls on the plates (DNA and RNA spike ins) yield detectable signals as expected (far right).

**Sample quality control using spike-ins (technical controls)**

It is important for any qPCR experiment to ensure that the quality of the input RNA is sufficiently high for effective amplification. The cDNA synthesis control (UniSp6) was added in the reverse transcription reaction giving the opportunity to evaluate the RT reaction. In addition to this, a DNA spike-in (UniSp3) is present on all panels. The DNA spike-in consists of a premixed combination of DNA template and primers. Deviations in this reaction will indicate inhibitions at the qPCR level. The level of these assays shown in the **Supplementary Figure 4** indicates that reverse transcription and qPCR were successful. The variation observed for UniSp6 is somewhat larger than what we usually see. Samples 8 and 9 yield stronger signals than the other samples, and the blank sample. This is not expected, but as these samples do not deviate for call rate or average Cq values, we do not expect this to be affecting the data set globally.


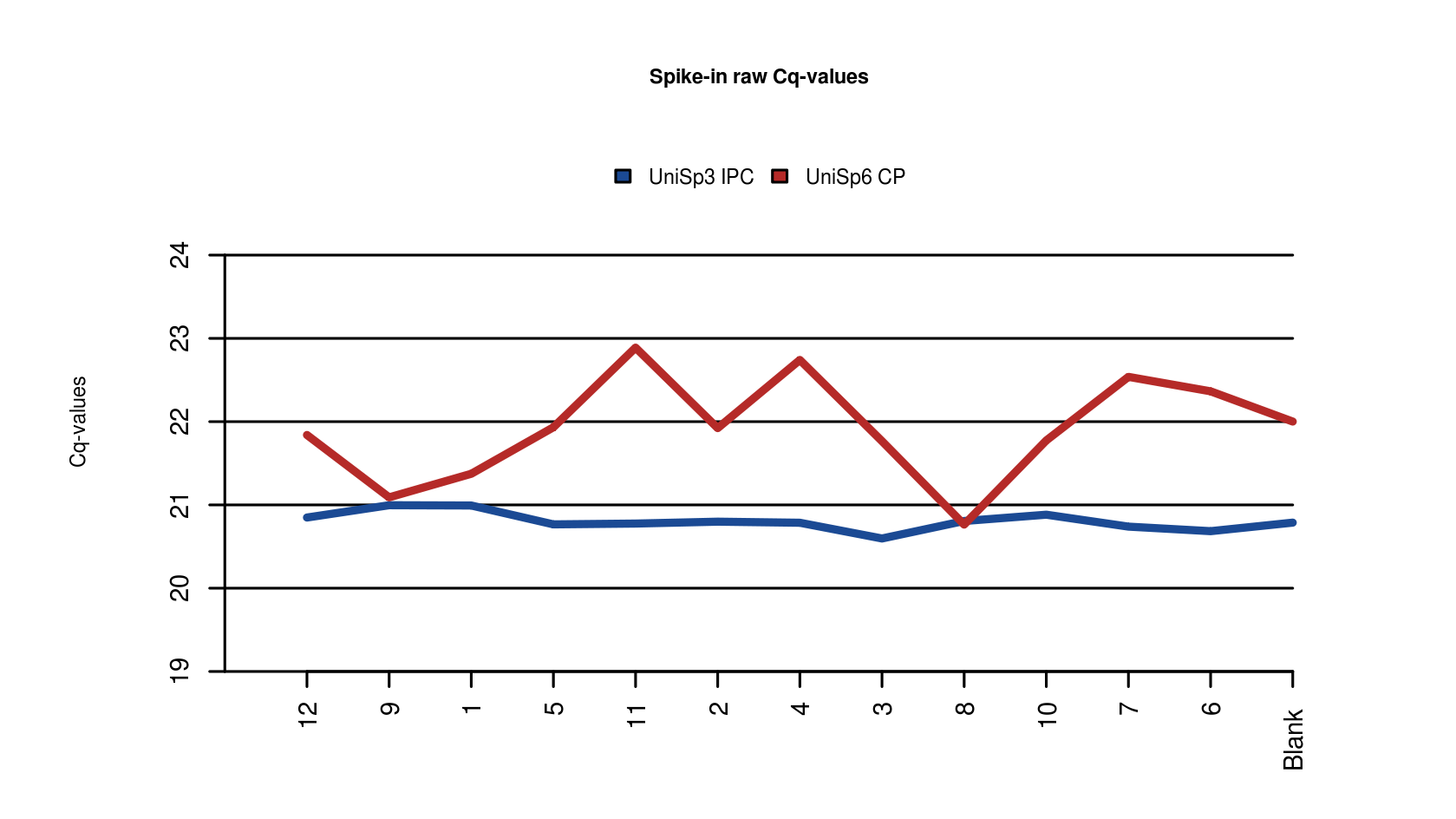


**Supplementary Figure 4**: The graph shows the raw Cqs obtained for the control assays.

**1.3 Data normalization**

Normalization is performed based on the average of the assays detected in all samples as this is shown to be the best normalization for qPCR studies involving numerous assays (Mestdagh, P. et.al. 2009). For the present study, this included 146 assays. The stability of the average of 146 microRNAs is higher than any single microRNA in the data set as measured by the NormFinder software (Andersen, C. et al, 2004).

The formula used to calculate the normalized Cq values are:

Normalized Cq = average Cq (n=12) – assay Cq (sample)

A higher value thus indicates that the microRNA is more abundant in the particular sample.

The normalized Cq values (dCq) are presented on the normalized data worksheet in the attached normalized_dcq_values.xlsx file.

**2. Next generation RNA sequencing**

RNA was isolated from MVs or cells using TRizol. Next generation sequencing was performed in an Illumina HiSeq 2500 (Illumina, San Diego, CA). Data normalization and analysis were conducted with the miARma-Seq too (Andres-Leon, E. et al, 2016). Briefly, sequence quality was assessed with FASTQC; sequence reads were aligned with Bowtie2 to the NCBI Rattus norvegicus annotation release 105 (Rnor_6.0). The resulting bam files were used by miARma-Seq for tabulating discovered miRNA. Reads were also aligned to Rfam 12.3 with Bowtie2 for categorization of RNA reads. To quantify the differential expression of miRNA between samples, the count of each miRNA was normalized as the percent of all discovered miRNAs in that sample. The fold-change was calculated as the percent expression of MV miRNA/Cell miRNA. KEGG Pathway enrichment analyses were performed based on the target genes of selected miRNAs.

**Supplementary Figure 5: Summary of the RNA-seq (reads)**

RC Cell


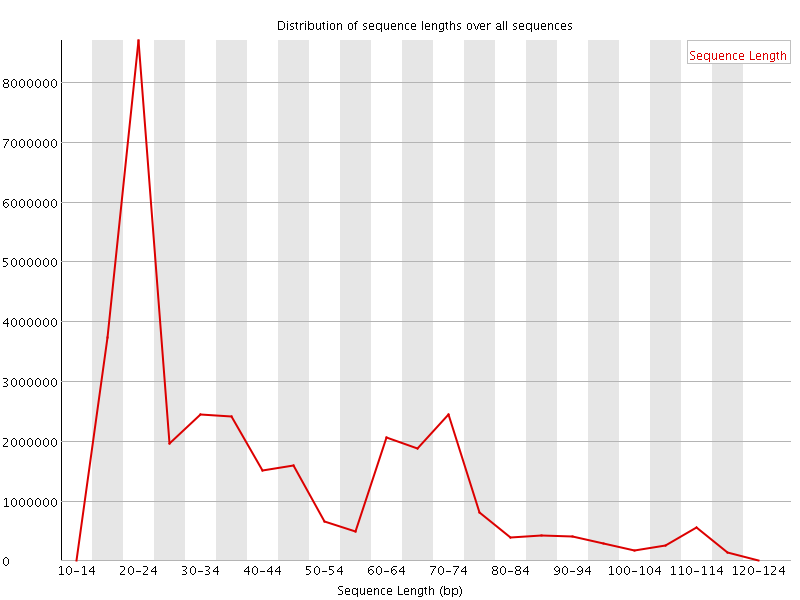


RC MV


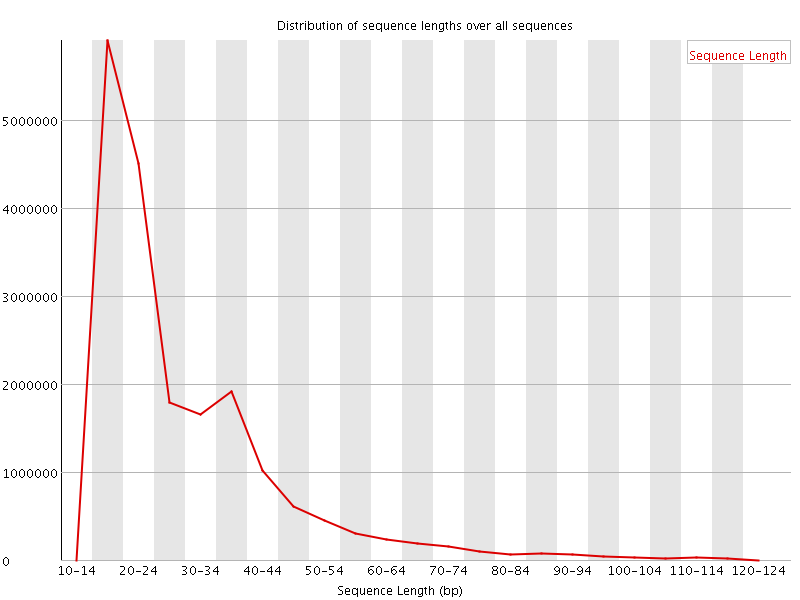


**Supplementary Figure 6: Quality of sequencing.**

RC Cell


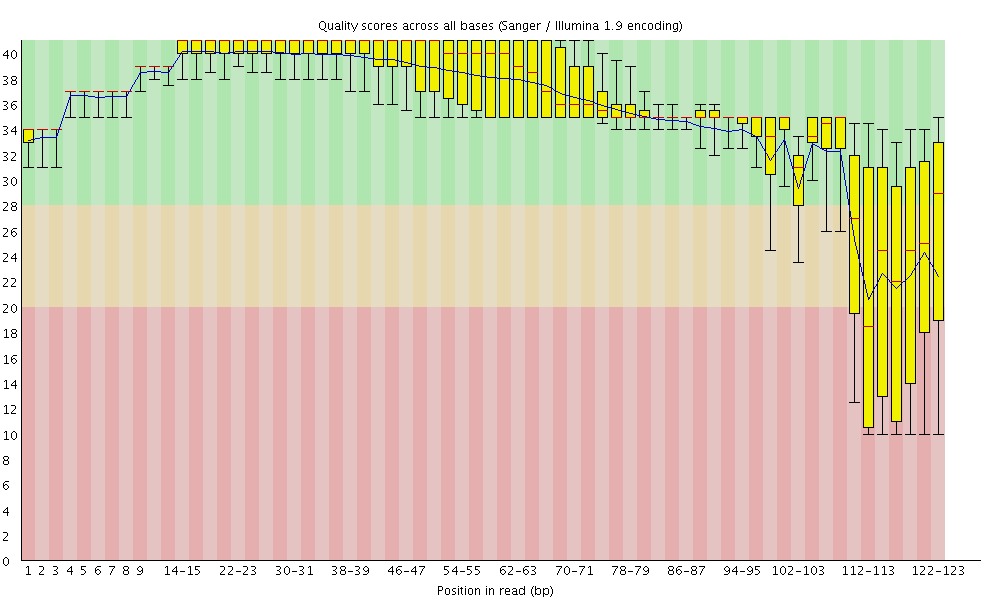


RC MV


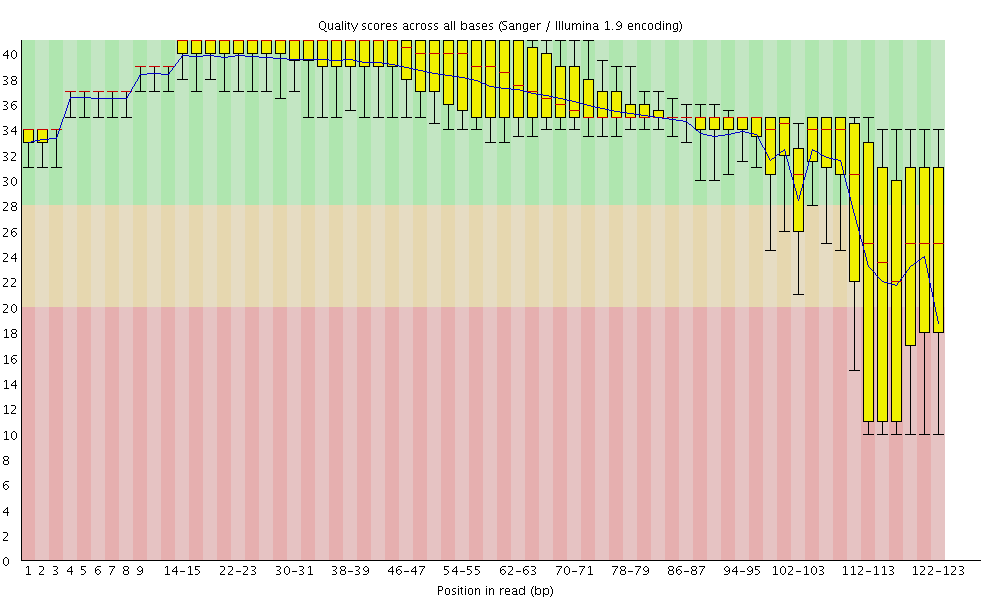


**3. Original full size gel images**

**Figure 2A:**

**
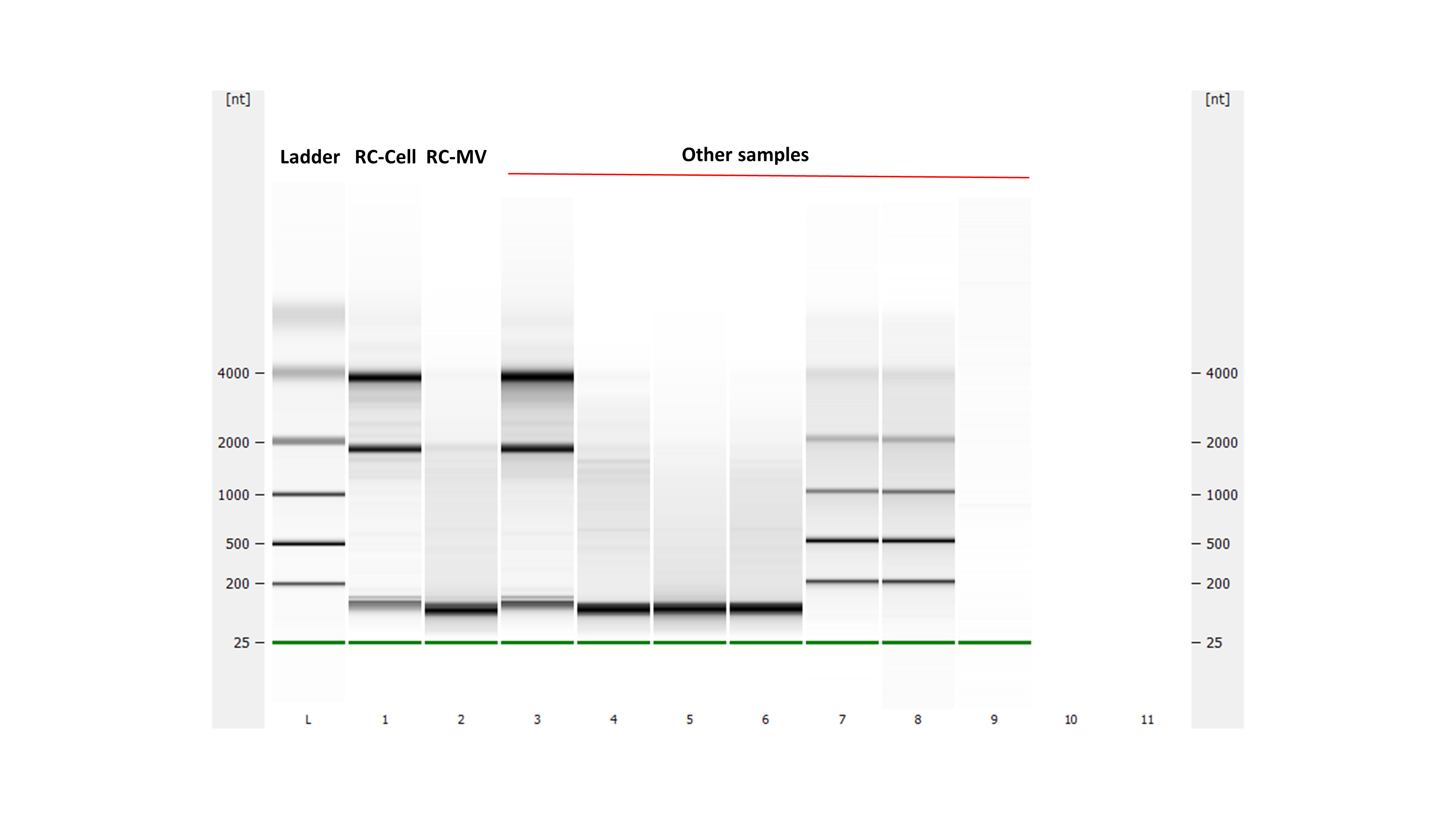
**

**Figure 2D:**

**
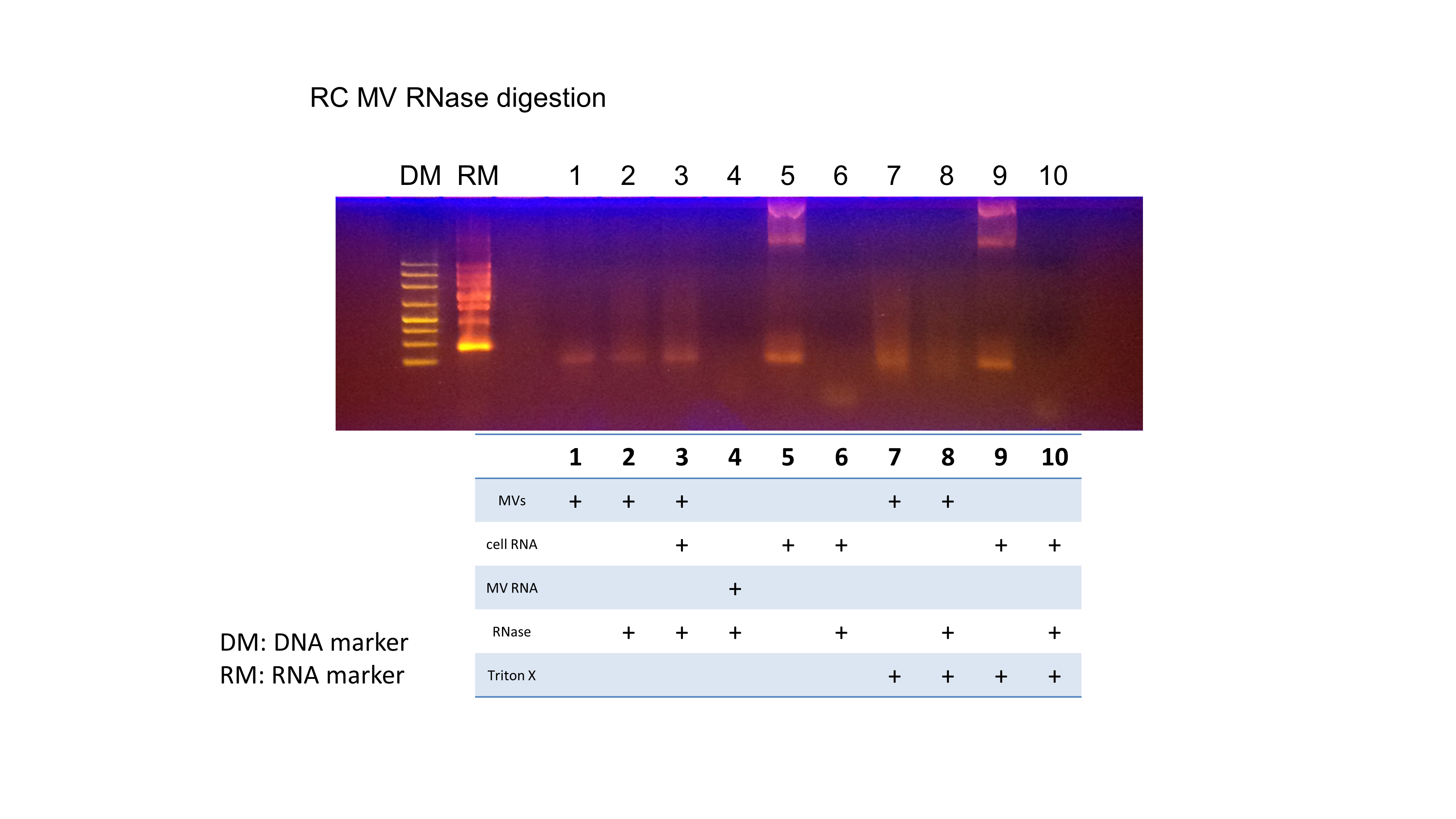
**

**Reference:**

Andersen, C. et al. (2004) Cancer Res., 64, 5245-5250

Andrés-León, E. et al. (2016) Sci. Rep., 6, 25749

Mestdagh, P. et al. (2009) Genome Biol.,10(6), R64
